# Clinical Study and Systematic Review of Pituitary Microadenomas vs. Macroadenomas in Cushing’s Disease: Does Size Matter?

**DOI:** 10.3390/jcm11061558

**Published:** 2022-03-11

**Authors:** Amit Akirov, Ilan Shimon, Maria Fleseriu, Idit Dotan, Yossi Manisterski, Nirit Aviran-Barak, Varda Nadler, Sandra Alboim, Tzipora Shochat, Gloria Tsvetov, Dania Hirsch

**Affiliations:** 1 Institute of Endocrinology, Rabin Medical Center Beilinson Hospital, Petach Tikva 4941492, Israel; i_shimon@netvision.net.il (I.S.); iditw78@yahoo.com (I.D.); gloria.tsvetov@gmail.com (G.T.); daniaron@netvision.net.il (D.H.); 2 Sackler School of Medicine, Tel Aviv University, Tel Aviv 6997801, Israel; 3 Pituitary Center and Departments of Medicine (Endocrinology) and Neurological Surgery, Oregon Health & Science University, Portland, OR 97239, USA; fleseriu@ohsu.edu; 4 Maccabi Health Care Services, Tel Aviv 6473925, Israel; manister_y@mac.org.il (Y.M.); aviran_n@mac.org.il (N.A.-B.); 5 Maccabi Health Care Services-Central Laboratory, Rehovot 7644620, Israel; nadler_v@mac.org.il (V.N.); alboim_s@mac.org.il (S.A.); 6 Statistical Consulting Unit, Rabin Medical Center, Beilinson Hospital, Petach Tikva 4941492, Israel; tzippysh@clalit.org.il

**Keywords:** corticotroph adenoma, Cushing’s disease, pituitary

## Abstract

**Background**: Reports on clinical and biochemical differences between adrenocorticotropic hormone (ACTH)-secreting pituitary microadenomas and macroadenomas are limited and inconsistent. **Objective:** Compare clinical and biochemical characteristics of patients with corticotroph microadenomas and macroadenomas and assess predictive factors for biochemical response to dynamic testing for Cushing’s disease (CD) in a clinical trial and a systematic review. A second aim was to evaluate differences between macroadenomas with and without cavernous and sphenoid sinus invasion. **Methods:** Retrospective charts review of patients with CD, treated at Rabin Medical Center between 2000 and 2020 or at Maccabi Healthcare Services in Israel between 2005 and 2017. Clinical and biochemical factors were compared between patients with corticotroph microadenomas and macroadenomas. We have also performed a systematic review of all studies (PRISMA guidelines) comparing corticotroph microadenomas with macroadenomas up to 31 November 2021. **Results**: The cohort included 105 patients (82 women, 78%; mean age, 41.5 ± 14.5 years), including 80 microadenomas (mean size, 5.2 ± 2.2 mm) and 25 macroadenomas (mean size, 18.0 ± 7.7 mm). Other baseline characteristics were similar between groups. Most common presentation suggestive for hypercortisolemia among patients with both micro- and macroadenomas were weight gain (46.3% vs. 48.0%, *p* = NS) and Cushingoid features (27.5% vs. 20.0%, *p* = NS). Mean 24 h urinary free cortisol (5.2 ± 5.4 × ULN vs. 7.8 ± 8.7 × ULN) and serum cortisol following low-dose dexamethasone (372.0 ± 324.5 vs. 487.6 ± 329.8 nmol/L), though higher for macroadenomas, were not significant. Levels of ACTH were greater for macroadenomas (1.9 ± 1.2 × ULN vs. 1.3 ± 0.8 × ULN, respectively, *p* = 0.01). Rates of recurrent/persistent disease were similar, as were rates of post-operative adrenal insufficiency and duration of post-operative glucocorticoid replacement. Macroadenomas with sphenoid or cavernous sinus invasion were associated with higher ACTH, 24 h free urinary cortisol, and serum cortisol following low-dose dexamethasone, compared with suprasellar or intrasellar macroadenomas. **Conclusions:** While ACTH-secreting macroadenomas exhibit higher plasma ACTH than microadenomas, there was no association between tumor size with cortisol hypersecretion or clinical features of hypercortisolemia. Though overall rare, increased awareness is needed for patients with CD with tumor extension in the cavernous or sphenoid sinus, which displays increased biochemical burden, highlighting that extent/location of the adenoma may be more important than size per se. Our systematic review, the first on this topic, highlights differences and similarities with our study.

## 1. Introduction

Cushing syndrome (CS) is caused by prolonged exposure to elevated levels of endogenous or exogenous glucocorticoids. Pituitary adenomas that secrete adrenocorticotropic hormone (ACTH) are derived from corticotroph cells in the anterior pituitary [1]. Pituitary hypersecretion of ACTH is the most common cause of endogenous CS [2,3]. Excess ACTH secreted by corticotroph cells is released into the circulation and acts on the adrenal cortex to produce hyperplasia and stimulate the secretion of adrenal steroids [4,5].

In most cases, ACTH-secreting pituitary adenomas are only a few mm in diameter, measuring on average 6 mm, and according to previous reports, only in less than 10% of cases, they are larger than 10 mm (macroadenoma) [6]. As with any other large pituitary mass, the larger pituitary adenomas may cause additional symptoms secondary to mass effect.

Previous studies, but not all, have shown that clinical presentation of patients with microadenomas is not significantly different, and there were no clinical signs or symptoms that differentiated patients with corticotroph macroadenomas from microadenomas [6,7,8,9].

The objective of our study was to provide an assessment of the clinical and biochemical characteristics of patients with corticotroph microadenomas and corticotroph macroadenomas and furthermore to compare patients with macroadenomas with cavernous and sphenoid sinus extension with those with sellar and suprasellar macroadenomas.

## 2. Methods

### 2.1. Patients and Setting

The study was conducted at Maccabi Healthcare Services, the second-largest publicly funded health maintenance organization (HMO) in Israel, and the Endocrine Institute of Rabin Medical Center, a large tertiary hospital in Israel. It was approved by the Ethics Review Boards of both facilities.

All patients referred to the Endocrine Institute of Rabin Medical Center and diagnosed with CD between 2000 and 2020 were included. The recruitment of the patients treated for CD at Maccabi Healthcare Services has been previously described [10]. The computerized database of the HMO was screened for all individuals who fulfilled the following criteria:

- The recorded biochemical data were compatible with a CD diagnosis as stipulated in the Endocrine Society Clinical Guidelines [11].
- A diagnosis of overt CD was established by an expert endocrinologist at the time of presentation.
- The diagnosis was retrospectively ascertained by the study authors at the time of data collection based on the documented biochemical and imaging tests, as well as management and follow-up details.

Histologic documentation of an ACTH-secreting tumor: in cases in which the histological reports were unavailable or inconclusive, biochemical and clinical resolution of the hypercortisolism after surgical resection was used as diagnostic confirmation.

- The results of second-line biochemical tests performed for the differential diagnosis of ACTH-dependent Cushing syndrome were retrieved and analyzed [7,8]. For each patient, the year in which the first elevated 24 h urinary free cortisol (UFC) result was recorded (2000–2017) was considered the time of diagnosis.

Postsurgical remission was defined as a low (<138 nmol/L) or undetectable serum cortisol level 18–24 h after the last dose of oral cortisone acetate or hydrocortisone during the first week of surgery; clinical adrenal insufficiency; the need for glucocorticoid replacement; disappearance of clinical features of hypercortisolism over the course of the follow–up; and/or normal UFC levels throughout the first year following the pituitary surgery. Recurrence was defined as the reappearance of clinical symptoms and signs of hypercortisolism, associated with abnormal screening tests for hypercortisolemia.

Measurements of all adenoma diameters were obtained by radiological examination of the pituitary, and tumors in which the largest diameter was at least 10 mm were considered to be macroadenomas. When no visible tumor was seen on magnetic resonance imaging (MRI) following a neuroradiologist review, the tumor was a microadenoma. 

Twenty-four-hour UFC was measured using a commercial radioimmunoassay (DiaSorin, Saluggia, Italy, or Siemens Healthcare Diagnostics).

The proliferative potential of corticotroph adenomas was investigated by the presence of Ki-67, a cell cycle antigen and marker of cell proliferation and aggression in various neoplasms [12]. The Ki-67 labeling index was expressed as a percentage of Ki-67-positive tumor nuclei per total tumor nuclei counted/specimen.

### 2.2. Study Procedure

The following data were collected by careful review of the patient files: demographic and clinical characteristics at diagnosis of CS, including age, sex, and body mass index; diagnosis of hypertension, diabetes mellitus or impaired fasting glucose, and osteoporosis; reason(s) for referral for Cushing syndrome screening tests; performance (yes/no) and results of diagnostic tests for Cushing syndrome, including UFC, morning cortisol level after overnight 1 mg dexamethasone, late-night salivary cortisol level, and dehydroepiandrosterone sulfate (DHEAS). Data regarding the clinical course after CD diagnosis were also retrieved: performance (yes/no) and time of surgery, medical therapy for hypercortisolism (yes/no), evidence of persistent/recurrent disease, time of disease recurrence, post-surgery glucocorticoid replacement administration (yes/no), and duration.

The Institutional Review Board of Rabin Medical Center and Maccabi Health Services approved the study.

### 2.3. Systematic Review

**Figure 1:**
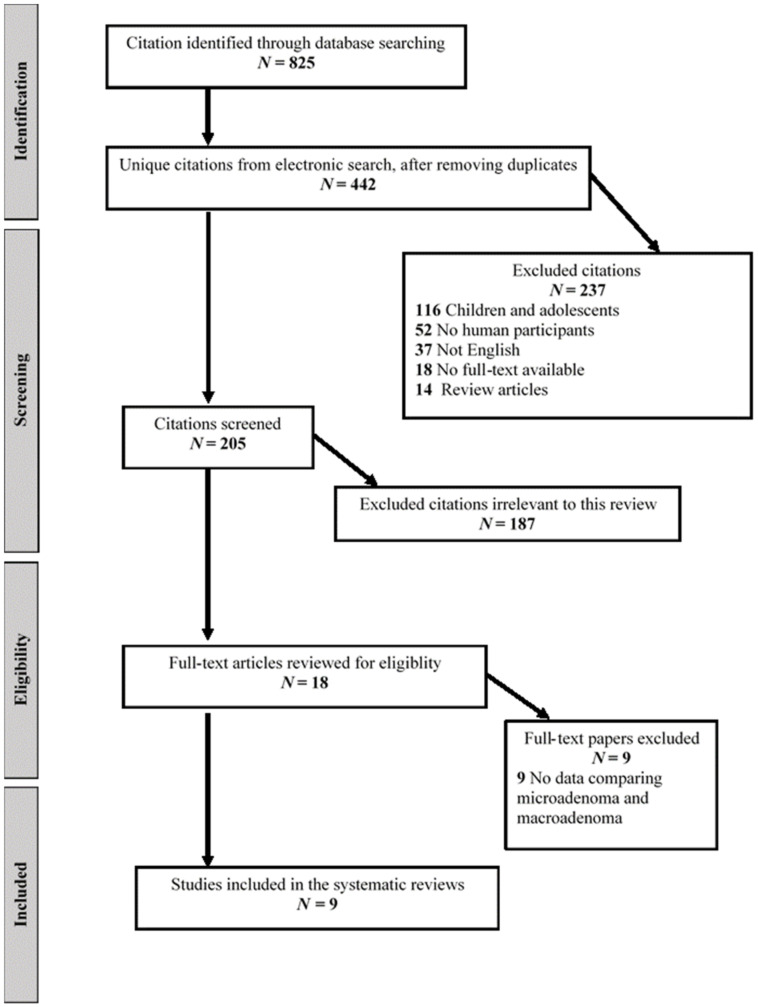
Systematic review flow diagram.

We performed a systematic review according to the Preferred Reporting Items for Systematic Reviews and Meta-Analyses (PRISMA) statement of studies [13]. The literature was searched from inception up to 31 December 2021 for relevant peer-reviewed articles written in English, using Medline Ovid, Medline (PubMed), Web of Science, and Google Scholar databases. The terms included in the search string were combined with Boolean operators as follows: ((“Cushing” OR “Corticotroph”) AND (“Pituitary”) AND (“Macroadenoma” OR “Size” OR “Diameter”)). We supplemented the electronic search by cross-referencing included papers, relevant sections of clinical practice guidelines, relevant systematic and narrative reviews. Two authors (A.A. and I.D.) conducted the search, and articles were first assessed by title, second by abstract, and third by full text. Another author (M.F.) reviewed all studies included. Original studies in adults ≥ 18 years of age with observational design (cross-sectional, case-control, and cohort) reporting data on clinical and biochemical characteristics of pituitary microadenomas vs. macroadenomas were included. Studies in children and adolescents, non-English articles, preprint articles, case reports, articles without pertinent data, non-research articles, and articles without full-text availability were excluded (Figure 1). The trial characteristics extracted included sample size and number of patients with macroadenoma and biochemical differences between patients with microadenoma and those with macroadenoma, including 24 h UFC, low dose and high dose DST, salivary cortisol, and ACTH.

### 2.4. Statistical Analysis

The statistical analysis was generated using SAS Software, Version 9.4 (SAS, Cary, NC, USA).

Continuous variables were presented by mean ± SD or median and IQR, and categorical variables were presented by (N,%); *t*-test was used to compare the value of continuous normal variables between study groups and Wilcoxon for skewed continuous variables, and Fisher’s exact test was used to compare the value of categorical variables between study groups. Two-sided *p* values less than 0.05 were considered statistically significant. Kruskal–Wallis test was used for non-normal continuous variables.

## 3. Results

### 3.1. Study Cohort

We identified 116 patients with CD. Following the exclusion of 11 patients with no data on pituitary MRI findings, the final study cohort consisted of 105 patients (82 women, 78%; mean age ± SD, 41.5 ± 14.5 years): 68 patients from the Maccabi Healthcare services clinics and 37 patients from the Endocrine Institute of Rabin Medical Center. Mean follow-up for the whole cohort was 5.7 ± 4.4 years.

Pituitary MRI revealed a microadenoma in 68 patients (64.8%) and a macroadenoma in 25 patients (23.8%). In 12 patients, no adenoma was visible on pituitary MRI (11.4%) and further investigation, including inferior petrosal sinus sampling (IPSS), confirmed a pituitary source for excess ACTH, thus were classified as having possible microadenomas. Of note, bilateral IPSS was completed for 18 patients, including those without a visible adenoma and patients with a small adenoma (<6 mm), and confirmed the diagnosis of pituitary source for hypercortisolism in all cases.

Baseline characteristics, as shown in Table 1, were similar between patients with ACTH-secreting microadenomas and macroadenomas, including age (mean age, 42.3 ± 14.9 vs. 39.0 ± 13.2 years, respectively), gender (women: 77.5% vs. 80.0%, respectively), body mass index (mean, 30.8 ± 7.5 vs. 29.4 ± 4.9 kg/m^2^, respectively), and prevalence of hypertension, osteoporosis/osteopenia, or menstrual irregularities. No significant difference was seen regarding disorders of glucose metabolism, but impaired fasting glucose or diabetes mellitus were slightly more common among those with microadenomas (Table 2).

Among both groups, the most common reason for completing an investigation for possible CS was weight gain: 37 patients (46.3%) with microadenomas and 12 patients (48.0%) with macroadenomas. Suspected Cushingoid features were the reason for investigation in more than a quarter of patients with microadenomas (27.5%) and in one of five patients with macroadenomas (20.0%). Virilization and/or oligomenorrhea were reported as the cause for investigation for nine patients (40.0%) with macroadenomas, compared with 15 patients (23.8%) with microadenomas.

### 3.2. Biochemical Data

**Figure 2:**
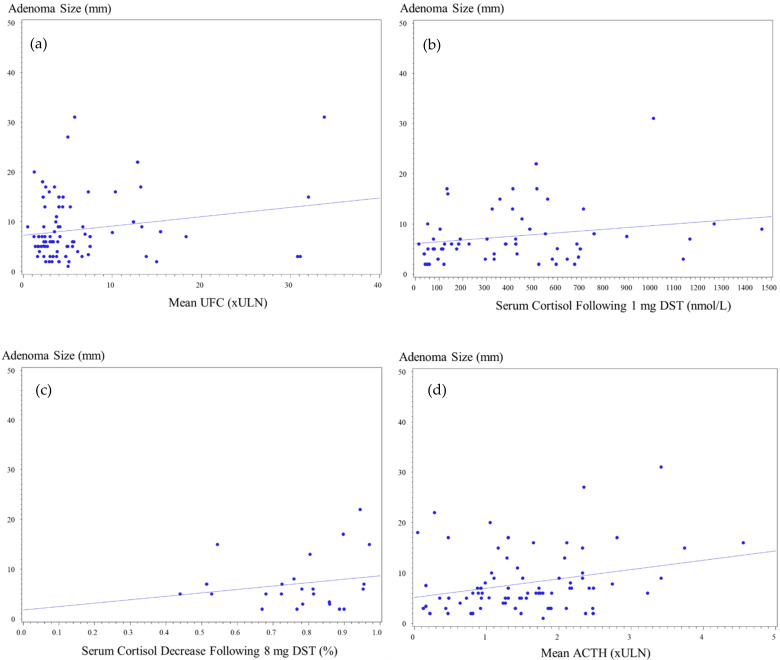
Correlation between adenoma size on MRI and biochemical profile (**a**) mean urinary free cortisol (UFC) (×ULN), *p* = 0.41; (**b**) serum cortisol after 1 mg dexamethasone suppression test (DST) (ng/mL), *p* = 0.09; (**c**) serum cortisol suppression after 8 mg DST (%), *p* = 0.5; (**d**) adrenocorticotropic hormone (×ULN), *p* = 0.03.

Data on UFC were available for 101 patients (96.2%), 78 (97.5%) patients with pituitary microadenoma and 23 (92.0%) with macroadenomas. Patients with microadenomas had UFC mean of 5.2 ± 5.4 × ULN (range, 0.5–31.1 × ULN), while in patients with macroadenoma, mean was 7.8 ± 8.7 × ULN (range, 1.2–33.8 × ULN) (*p* = 0.13). UFC levels > 4 × ULN were recorded in 31 patients (39.7%) with microadenoma and in 12 patients (52.2%) with macroadenoma (*p* = NS). Mean UFC levels < 2 × ULN were reported in one patient with a macroadenoma (4.0%), compared with 11 patients (14.1%) with microadenomas. Analysis of the maximal UFC levels indicated higher values for patients with macroadenomas (mean, 8.6 ± 8.7 × ULN; range, 1.2–34.7 × ULN) than microadenomas (6.2 ± 5.5 × ULN; range, 0.8–32.2 × ULN), but the difference was not statistically significant (*p* = NS) (Figure 2).

Data on 1 mg overnight DST results were available for 69 patients: 55 (74.3%) patients with microadenomas and 14 (56.0%) with macroadenomas, with abnormal serum cortisol levels following low-dose dexamethasone reported for 94.2% (65 of 69 patients) of the whole cohort. Mean serum cortisol levels following low-dose DST were higher among patients with macroadenomas than microadenomas (487.6 ± 329.8 vs. 372.0 ± 324.5 nmol/L, respectively; *p* = 0.19), but there was an overlap between ACTH-secreting microadenomas (range, 14–1461 nmol/L) and macroadenomas (range, 53–1260 nmol/L). Normal results, defined as serum cortisol < 50 nmol/L following 1 mg dexamethasone, were recorded for four patients with microadenomas but for none of those with macroadenomas (7.3% vs. 0%, respectively). Mildly elevated serum cortisol levels following DST, defined as serum cortisol between 50 and 138 nmol/L, were reported in 14 patients with a microadenoma, compared with three patients with a macroadenoma (25.4% vs. 21.4%, respectively). Serum cortisol levels > 138 nmol/L were reported in most patients with microadenomas or macroadenomas (67.3% vs. 78.6%, respectively) (Figure 2).

Late-night salivary cortisol results were available only for 14 patients, with elevated levels reported in all three patients (100%) with macroadenoma and in 9 out of 11 patients with microadenomas (81.8%).

Serum ACTH levels were available for 93 patients: 74 (92.5%) with micro and for 19 (76.0%) with macroadenomas. Mean ACTH was 1.3 ± 0.8 × ULN for microadenomas (range, 0.1–3.4 × ULN), compared with a mean of 1.9 ± 1.2 × ULN among those with macroadenomas (range, 0.3–4.6 × ULN) (*p* = 0.03). Sixteen out of nineteen (84%) patients with macroadenomas and available data on ACTH concentrations had serum ACTH levels above the normal range, compared with 46 out of 74 (62%) patients with microadenomas. ACTH levels > 2 × ULN were recorded more frequently in patients with macroadenomas than microadenomas (53.0% vs. 20.0%, respectively) (Figure 2).

Overnight 8 mg DST results were available for 29 patients, including 24 patients with microadenomas and 5 patients with macroadenomas. The degree of cortisol suppressibility was similar for macroadenomas and microadenomas (83.1 ± 17.3% vs. 73.7 ± 20.2%, respectively). All five patients (100%) with macroadenoma had >50% reduction in plasma cortisol, as did 22 out of 24 (81.5%) patients with microadenoma (Figure 2).

Data on plasma DHEAs concentrations were available for 50 patients: 44 patients with microadenomas and 6 patients with macroadenomas; mean DHEAs levels were similar for both groups (mean, 8.95 ± 19.4 ng/mL vs. 8.94 ± 6.3 ng/mL, respectively).

### 3.3. Macroadenoma Extension and Biochemical Profile

**Figure 3:**
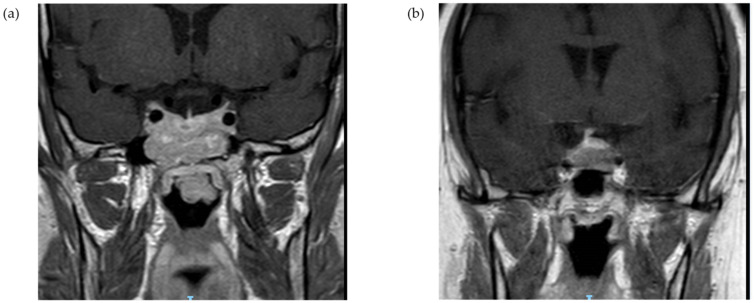
Illustrative radiographic MR imaging. Coronal T1, non-contrast (**a**) suprasellar and infrasellar lesion with a large exophytic portion invading into the nasopharynx, involving the clivus and the sphenoidal sinus (Patient #6); (**b**) a suprasellar lesion invading into the left cavernous sinus (Patient #12).

Among patients with corticotroph macroadenoma, data on the radiographic characteristics were available for 23 out of 25 tumors. Suprasellar extension was reported for 15 patients (65.2%) with optic chiasm compression in six cases (26.1%). Cavernous sinus invasion was evident in 10 cases (43.5%), and sphenoid sinus invasion was documented for five patients (21.7%) (illustrative MR imaging, Figure 3). From a clinical standpoint, six patients (26.1%) reported headaches, and five patients (20.0%) had visual complaints, including two patients with bitemporal hemianopsia (Table 3). Median UFC values were highest for tumors with sphenoid sinus invasion (median, 5.8 × ULN; IQR, 3.1–23.5 × ULN) or cavernous sinus invasion (median, 5.8 × ULN; IQR, 3.2–11.6 × ULN), followed by tumors with suprasellar extension with no cavernous sinus invasion (median, 4.4 × ULN; IQR, 2.3–11.0 × ULN) or intrasellar macroadenomas (median, 4.6 × ULN; IQR, 3.6–8.8 × ULN) (*p* = 0.67). Urinary free cortisol levels >4 × ULN were reported for four out of five patients (80%) with sphenoid sinus invasion and for six out of ten patients (60%) with cavernous sinus invasion, compared with four out of eight (50%) patients with suprasellar extension with no cavernous sinus invasion and two out of four patients (50%) with intrasellar tumors. Similarly, serum cortisol levels following low-dose DST were higher among patients with tumors invading the sphenoid sinus (reported in two patients: 513 and 1004 nmol/L) or the cavernous sinus (reported in three patients: 410, 509, and 1004 nmol/L), compared with intrasellar tumors (median, 324 nmol/L; IQR, 93–984 nmol/L) (*p* = 0.57). Furthermore, median ACTH concentrations were highest for patients with sphenoid sinus invasion (median, 2.0 × ULN; IQR, 1.2–3.1 × ULN), or cavernous sinus invasion (median, 2.0 × ULN; IQR, 1.0–2.3 × ULN), followed by the suprasellar extension (median, 1.7 × ULN; IQR, 0.9–2.3 × ULN), and lowest for those with intrasellar macroadenomas (median, 0.5 × ULN; IQR, 0.3–1.9 × ULN) (*p* = 0.32).

### 3.4. Surgical Treatment Outcomes

Most patients in both groups underwent transsphenoidal surgery, including 71 out of 74 (96.0%) with microadenomas and 23 out of 25 (92.0%) with macroadenomas. Data on the Ki-67 proliferation index were available for 15 patients with microadenomas and for 9 with macroadenoma; mean of 3.7 ± 3.1% vs. 5.7 ± 4.8%, respectively. Ki-67 labeling index ≥5% was reported for six out of nine macroadenomas (66%), compared with six out of fifteen microadenomas (40%).

Postoperatively, rates of persistent or recurrent disease following surgery were 30.4% (7 out of 23 patients) among patients with macroadenomas (mean follow-up period of 7.3 ± 4.6 years), compared with rates of 35.2% (25 out of 71 patients) among those with microadenomas (mean follow-up period of 5.1 ± 4.1 years) (*p* = NS). The persistent disease was reported for three patients (12.0%) with a macroadenoma, compared with 19 patients (26.8%) with a microadenoma. Recurrent disease was reported for five (21.7%) and six (8.5%) patients, respectively (*p* = NS). Four out of twelve patients (33%) with no visible adenoma on MRI had persistent disease following surgery. The mean time to recurrence after surgery was similar for microadenomas and macroadenomas (43.0 ± 34.6 months vs. 45.3 ± 25.2 months, respectively).

Data on glucocorticoid replacement following surgery were available for 18 patients with macroadenomas, of whom 15 (83.3%) required treatment, as did 52 out of 62 (83.9%) patients with microadenomas. The mean duration of glucocorticoid replacement following surgery was similar for macroadenomas and microadenomas (9.7 ± 11.9 months vs. 9.4 ± 8.3 months, respectively).

### 3.5. Systematic Review

As detailed in the flow diagram (Figure 1), we retrieved a total of 825 citations from our electronic searches, ultimately yielding 442 unique citations after removing any duplicates. References from the hand search were all included in the electronic database searches. We reviewed 18 full-text papers for eligibility, and nine trials published between 1998 and 2021 met the inclusion criteria and were included in the systematic review [6,7,8,14,16,17,18,19,20]. We excluded trials with no available data on the biochemical profile of corticotroph microadenoma vs. macroadenoma. A summary of trials included in the systematic review is shown in Table 3. Of the included studies, four were conducted in Europe [6,8,14,19], two in the United States [7,17], two in Asia [9,20], and one study in Brazil [18]. The number of participants ranged from 7 to 74 patients with corticotroph macroadenoma [6,7,8,14,16,17,18,19,20].

Data on UFC were not available in three studies [6,14,19], but five studies reporting on UFC showed no difference in levels between microadenoma and macroadenoma [7,8,16,17,19], and only Machado et al. reported on significant difference in UFC, with higher levels reported for patients with microadenoma [18]. Four studies provided data on cortisol levels following 1 mg dexamethasone with no difference in all studies between microadenoma and macroadenoma [6,17,18,19]. Data on serum cortisol levels following high dose DST were available from seven studies, three reporting higher serum cortisol among patients with macroadenoma [6,7,8] and four suggesting no difference between patients with microadenomas and macroadenomas [9,17,18,19]. All studies provided data on ACTH levels, with seven studies showing higher levels for macroadenomas [6,7,8,14,18,19,20], but two studies reported no difference in ACTH levels between the groups [9,17] (Table 3).

## 4. Discussion

We show here in the first systematic review and confirmed by our study that patients with Cushing’s disease secondary to ACTH-secreting microadenomas and macroadenomas present with similar clinical and biochemical characteristics, except for higher plasma ACTH concentrations among those with corticotroph macroadenomas. This suggests there is a lack of association between tumor size per se with cortisol secretion values or clinical characteristics. Tumor location or extension may be more important than merely tumor size, as tumors invading the sphenoid or cavernous sinus were associated with both higher plasma ACTH and cortisol secretion.

More than two decades ago, Katznelson et al. [7] and Selvais et al. [8] compared corticotroph macro with microadenomas and suggested that macroadenomas were less suppressible after 1 mg dexamethasone administration and after 48 h high dose dexamethasone suppression test and that decrease in UFC post dexamethasone was lower among patients with macroadenoma. Furthermore, patients with macroadenoma were more likely to have baseline plasma ACTH concentrations above the normal range, and ACTH values were higher in patients with macro compared with those with microadenomas [6]. On the other hand, a more recent study showed no significant differences in plasma ACTH, serum cortisol, or UFC between patients with ACTH-secreting microadenomas and macroadenomas [9] (Table 3).

Differences in ACTH assays over time could also influence these results [16,17,19,20].

In our study, patients with corticotroph microadenomas and macroadenomas shared similar clinical features, with no difference in rates of hypertension, osteoporosis, impaired fasting glucose, or diabetes mellitus at presentation. Interestingly, there was no significant age or gender difference between groups, with a female predominance in both groups. Furthermore, weight gain and Cushingoid features were the most common reasons for completing an investigation for Cushing’s syndrome in both groups. Prior studies to assess clinical differences between microadenomas and macroadenomas patients showed inconsistent data [9], but in most series, rates of hypertension, diabetes, osteoporosis, and BMI were similar in both groups [8,15,16,17,18].

Previous studies [7,8] suggested that patients with macroadenomas were more likely to have baseline plasma ACTH concentrations above the normal range (83% vs. 45%). Woo et al. [6] similarly showed plasma ACTH values were higher for 18 patients with corticotroph macro vs. 183 with microadenomas. In line with these previous reports and others [6,7,8,14,16,17] but in contrast with other series [9,15], our study shows higher plasma ACTH among patients with corticotroph macroadenoma, compared with microadenomas, and likewise, more patients with baseline plasma ACTH values above the normal range in macroadenoma patients, compared with microadenoma ones (84% vs. 62%). The correlation between adenoma size and the severity of hormonal hypersecretion is well-established for prolactin-secreting tumors, and our findings support a linear correlation between ACTH and maximum adenoma diameter. It has been postulated for other pituitary adenomas that a correlation between hormonal hypersecretion and tumor size is due to the fact that the vast majority of cases are secondary to a monoclonal adenoma, composed of an identical histological type [21].

Unlike the association between tumor size with plasma ACTH concentrations, no association was seen with UFC, low-dose DST, or high-dose DST. It should be noted that while serum cortisol following low- or high-dose DST was not significantly different between groups, all false-negative results of low-dose DST were documented among patients with microadenoma, while all patients with macroadenomas had abnormal results. Furthermore, more than 80% of patients with macroadenomas had significantly increased serum cortisol levels following DST (>138 nmol/L), while two-thirds of patients with microadenomas had only mildly elevated serum cortisol levels (50–138 nmol/L). These findings suggest that low-dose DST may perform better in corticotroph macroadenomas than in microadenomas, and a positive low DST should be followed by additional confirmatory testing. However, contrary to prior reports showing reduced suppressibility during high dose DST in macroadenomas, in our study, all patients with macroadenoma who underwent high dose DST had >50% reduction in plasma cortisol. High-dose DST has limited value in diagnosis overall [1,22], but our data do not support the theory that corticotroph macroadenomas are less suppressible after 8 mg dexamethasone administration [6,7,8,14]. However, the limited number of patients with a macroadenoma in our study precludes any definitive conclusion.

Surprisingly, our data show that patients with corticotroph macroadenomas exhibit lower cortisol secretion for the degree of ACTH elevation. While it is possible that the lack of difference is secondary to the limited sample size, there are several other potential explanations. First, large pituitary adenomas may be less differentiated and less efficient at the processing of proopiomelanocortin to ACTH, resulting in the production of biologically less potent ACTH precursors and fragments that may cross-react in the ACTH assay [23]. Undoubtedly, a liquid chromatography-tandem mass spectrometry (LC-MS/MS) assay for biologically active intact ACTH can provide a more precise estimation of ACTH values [24]. Second, while mean ACTH concentrations were significantly higher among patients with macroadenomas, there was a significant overlap of ACTH values between the groups, suggesting that large tumors may produce a similar amount of ACTH as small tumors; thus, these findings may be partly due to low level of hormone production per tumor mass or due to disturbances in the regulated exocytotic pathway [25]. Third, variations in ACTH pulsatility and action on the adrenal gland may affect cortisol secretion [15].

Interestingly, we found for the first time that tumors with sphenoid or cavernous sinus invasion were associated with higher levels of ACTH, UFC, and serum cortisol following low-dose DST, compared to patients with suprasellar or intrasellar macroadenomas, suggesting that tumor location may have a more significant impact on the biochemical profile of patients with CD. However, the limited sample size prohibited statistical analysis.

Surgical remission rates were similar in patients with microadenomas and macroadenomas, similar to other series [9,26], ranging between 65% and 70%, also in line with previous reports [27]. These findings suggest that surgical intervention success is not dependent merely on tumor size and that tumor invasion and surgical expertise are more important factors in achieving remission.

While there was a trend for a higher Ki-67 labeling index in corticotroph macroadenomas (5.7 ± 4.8% vs. 3.7 ± 3.1%, respectively), the difference was not statistically significant, although Ki-67 labeling index ≥5% was more common among macroadenoma patients. As suggested by Losa and colleagues, it is possible that the difference in the proliferation activity is the main factor underlying the different patterns of growth among corticotroph microadenoma and macroadenomas [14]. Despite last WHO recommendations [28] suggesting no need to measure Ki-67 for pituitary adenomas, it might be useful to continue it for assessing corticotroph macroadenomas prognosis.

While previous studies have investigated the clinical and biochemical differences between microadenomas and macroadenomas in patients with Cushing’s disease, the data are limited and inconsistent. Our systematic review identified nine previous studies on this topic; six of these did not provide any data on UFC levels, three of these reports were published more than 20 years ago, and most studies included less than 20 patients with macroadenoma. Our study provides a more up-to-date assessment and takes into account advances in imaging technologies and laboratory testing. Moreover, our cohort included a relatively large number of patients with macroadenoma, with data on UFC levels for almost all patients.

Our study had several important limitations, including the retrospective design, missing or unavailable biochemical data as only 68 patients had no missing values in the main hormonal variables (ACTH, serum cortisol after 1 mg DST, and UFC), fewer patients with macroadenoma, as well as low number of patients who underwent cortisol salivary testing. However, we have more patients with macroadenomas compared with previous reports, which may be explained by our stricter exclusion criteria (e.g., unspecified hypercortisolism, more likely to harbor microadenomas). A potential referral bias, as those who have larger tumors are more likely to be referred to our specialized center, could also play a role; however, the other studies were also done in pituitary centers. In addition, a meta-analysis was not performed due to the heterogeneity of the studies included in terms of patients recruited, imaging modalities, laboratory techniques, and outcomes reported.

Our study suggests classifying corticotroph tumors solely according to the tumor size may not be as clinically important as is tumor invasion; the latter is impacting the potential for complete tumor removal and biochemical remission. Furthermore, a low threshold for CS screening in patients with all macroadenomas is also very important as patients with larger adenomas can sometimes present with mild hypercortisolism.

## Figures and Tables

**Table 1:** Baseline characteristics. Baseline characteristics and comorbidities of patients by each category. * As data on signs and symptoms were not available for all patients, data presented as number of patients with the specific feature of all relevant patients with available data. ^#^ *p* < 0.05. UFC—urinary free cortisol; DST—dexamethasone suppression test; ACTH—adrenocorticotropic hormone; DM—diabetes mellitus; SD—standard deviation; ULN—upper limit of normal.

	*Microadenoma (<10 mm)*	*Macroadenoma (≥10 mm)*
*Patients, n*	80	25
*Female, n (%)*	62 (77.5%)	20 (80.0%)
*Age (years), mean ± SD*	42.3 ± 14.9	39.0 ± 13.2
*Adenoma size (mm), mean ± SD*	5.2 ± 2.1	18.0 ± 7.7
*Body mass index (kg/m2), mean ± SD*	30.8 ± 7.5	29.4 ± 4.9
*Hypertension, n (%)*	40 (50.0%)	11 (44.0%)
*IFG/DM*	36 (45.0%)	8 (32.0%)
*Osteoporosis/osteopenia*	19/45 (42.2%) *	7/16 (43.8%) *
*Menstrual irregularities*	31/43 (71.1%) *	9/13 (69.2%) *
*UFC (mean ± SD)*	5.2 ± 5.4 × ULN	7.8 ± 8.7 × ULN
*Serum cortisol post 1 mg DST (mean, nmol/L ± SD)*	372.0 ± 324.5	487.6 ± 329.8
*Serum ACTH (mean ± SD)*	1.3 ± 0.8 × ULN ^#^	1.9 ± 1.2 × ULN ^#^
*Suprasellar extension (n, %)*	4/76 (5.2%)	14/23 (60.8%)
*Cavernous sinus invasion*	2/76 (2.6%)	10/23 (43.5%)

**Table 2:** Corticotroph macroadenomas. Biochemical and radiographic characteristics of corticotroph macroadenomas. Two patients with macroadenomas are not included due to missing radiographic data on tumor extension. UFC—urinary free cortisol; DST—dexamethasone suppression test; ACTH—adrenocorticotropic hormone.

Pt	Sex	Age	Size (mm)	Intrasellar	Suprasellar Extension	Cavernous Sinus Invasion	Optic Chiasm Compression	Sphenoid Sinus Invasion	Headache	Visual Complaints	UFC (mean × ULN)	ACTH (mean × ULN)	1 mg DST (nmol/L)	Persistent or Recurrent	
**1**	F	32	10	1	0	0	0	0	0	0	3.7	1.1	53	NA	0
**2**	F	32	31	0	0	1	0	1	0	0	5.8	NA	NA	NA	0
**3**	M	32	31	0	0	1	0	1	0	0	33.8	3.4	1004	NA	0
**4**	F	24	27	0	1	1	1	1	1	0	5.0	2.3	NA	NA	1
**5**	M	30	22	0	1	1	0	0	1	0	12.8	0.3	509	94%	0
**6**	M	38	44	0	0	1	0	1	0	0	1.2	1.0	NA	NA	1
**7**	F	42	18	0	1	0	1	0	1	1	2.1	0.0	NA	NA	0
**8**	M	25	17	0	1	0	0	1	0	0	13.2	2.8	513	NA	0
**9**	F	23	17	0	1	0	0	0	0	0	3.5	0.5	133	90%	0
**10**	F	53	17	0	1	0	1	0	1	1	2.5	1.3	412	NA	1
**11**	F	56	16	0	1	0	0	0	1	0	2.9	4.6	136	NA	0
**12**	F	21	16	0	1	1	0	0	0	0	10.3	2.1	NA	NA	0
**13**	F	57	16	0	1	1	1	0	0	0	7.3	1.6	NA	NA	0
**14**	F	53	15	0	1	0	1	0	0	0	4.5	1.2	558	NA	0
**15**	F	36	15	0	1	0	0	0	0	0	2.2	2.3	357	97%	0
**16**	F	46	15	0	1	0	0	0	1	1	32.0	3.7	NA	54%	0
**17**	F	13	15	0	0	1	0	0	0	0	4.0	NA	NA	NA	1
**18**	F	28	13	0	1	1	0	0	0	1	2.4	2.1	410	NA	1
**19**	F	43	13	1	0	0	0	0	0	0	4.0	1.3	324	NA	0
**20**	F	46	13	0	1	0	0	0	0	0	4.4	NA	NA	80%	0
**21**	F	66	13	1	0	0	0	0	0	0	5.3	NA	708	NA	1
**22**	F	36	10	1	0	0	0	0	0	0	12.4	2.3	1260	NA	0
**23**	M	52	42	0	1	1	1	0	0	1	NA	NA	NA	NA	1

**Table 3:** Systematic review: summary of available data. UFC—24 h urinary free cortisol; DST—dexamethasone suppression test; ACTH—adrenocorticotropic hormone; NA—not available.

Study	Patients	UFC	Plasma Cortisol Following Low-Dose DST	Plasma Cortisol Following High-Dose DST	Late-Night Salivary Cortisol	ACTH
Selvais et al., 1998 [8]	21 patients (macroadenoma: 11 patients)	No difference	NA	Macroadenoma > Microadenoma	NA	Macroadenoma > Microadenoma
Katznelson et al., 1998 [7]	44 patients (macroadenoma: 20 patients)	No difference	NA	Macroadenoma > Microadenoma	NA	Macroadenoma > Microadenoma
Losa et al., 2000 [14]	51 patients (macroadenoma: 15 patients)	NA	NA	NA	NA	Macroadenoma > Microadenoma
Woo et al., 2005 [6]	201 patients (macroadenoma: 18 patients)	NA	NA	Macroadenoma > Microadenoma	NA	Macroadenoma > Microadenoma
Hwang et al., 2009 [9]	30 patients (macroadenoma: 7 patients)	No difference	NA	No difference	NA	No difference
Mathioudakis et al., 2012 [15]	53 patients (macroadenoma: 16 patients)	No difference	No difference	No difference	No difference	No difference
Machado et al., 2016 [16]	317 patients (macroadenoma: 74 patients)	Microadenoma> Macroadenoma	No difference	No difference	No difference	Macroadenoma > Microadenoma
Witek et al., 2016 [17]	59 patients (macroadenoma: 20 patients)	No difference	No difference	NA	NA	Macroadenoma > Microadenoma
Walia et al., 2021 [18]	190 patients (macroadenoma: 46 patients)	NA	No difference	No difference	No difference	Macroadenoma > Microadenoma

## Data Availability

The data presented in this study are available on request from the corresponding author.

## References

[1] Fleseriu M., Auchus R., Bancos I., Ben-Shlomo A., Bertherat J., Biermasz N.R., Boguszewski C.L., Bronstein M.D., Buchfelder M., Carmichael J.D. (2021). Consensus on diagnosis and management of Cushing’s disease: A guideline update. Lancet Diabetes Endocrinol..

[2] Lacroix A., Feelders R.A., Stratakis C.A., Nieman L.K. (2015). Cushing’s syndrome. Lancet.

[3] Melmed S. (2020). Pituitary-Tumor Endocrinopathies. N. Engl. J. Med..

[4] Cushing H. (1932). The basophil adenomas of the pituitary body and their clinical manifestation. Bull. Johns Hopkins Hosp..

[5] Newell-Price J., Bertagna X., Grossman A.B., Nieman L.K. (2006). Cushing’s syndrome. Lancet.

[6] Woo Y.S., Isidori A.M., Wat W.Z., Kaltsas G.A., Afshar F., Sabin I., Jenkins P.J., Monson J.P., Besser G.M., Grossman A.B. (2005). Clinical and Biochemical Characteristics of Adrenocorticotropin-Secreting Macroadenomas. J. Clin. Endocrinol. Metab..

[7] Katznelson L., Bogan J.S., Trob J.R., Schoenfeld D.A., Hedley-Whyte E.T., Hsu D.W., Zervas N.T., Swearingen B., Sleeper M., Anne K. (1998). Biochemical Assessment of Cushing’s Disease in Patients with Corticotroph Macroadenomas. J. Clin. Endocrinol. Metab..

[8] Selvais P., Donckier J., Buysschaert M., Maiter D. (1998). Cushing’s disease: A comparison of pituitary corticotroph microadenomas and macroadenomas. Eur. J. Endocrinol..

[9] Hwang Y.-C., Chung J.H., Min Y.-K., Lee M.-S., Lee M.-K., Kim K.-W. (2009). Comparisons between Macroadenomas and Microadenomas in Cushing’s Disease: Characteristics of Hormone Secretion and Clinical Outcomes. J. Korean Med. Sci..

[10] Hirsch D., Tsvetov G., Manisterski Y., Aviran-Barak N., Nadler V., Alboim S., Kopel V. (2017). Incidence of Cushing’s syndrome in patients with significant hypercortisoluria. Eur. J. Endocrinol..

[11] Nieman L.K., Biller B.M.K., Findling J.W., Newell-Price J., Savage M.O., Stewart P.M., Montori V. (2008). The Diagnosis of Cushing’s Syndrome: An Endocrine Society Clinical Practice Guideline. J. Clin. Endocrinol. Metab..

[12] Saeger W., Koch A. (2021). Clinical Implications of the New WHO Classification 2017 for Pituitary Tumors. Exp. Clin. Endocrinol. Diabetes.

[13] Moher D., Liberati A., Tetzlaff J., Altman D.G., The PRISMA Group (2009). Preferred reporting items for systematic reviews and meta-analyses: The PRISMA statement. PLoS Med..

[14] Losa M., Barzaghi R.L., Mortini P., Franzin A., Mangili F., Terreni M.R., Giovanelli M. (2000). Determination of the Proliferation and Apoptotic Index in Adrenocorticotropin-Secreting Pituitary Tumors: Comparison between Micro- and Macroadenomas. Am. J. Pathol..

[15] Mathioudakis N., Pendleton C., Quiñones-Hinojosa A., Wand G.S., Salvatori R. (2011). ACTH-secreting pituitary adenomas: Size does not correlate with hormonal activity. Pituitary.

[16] Machado M., Alcantara A., Pereira A., Cescato V., De Castro Musolino N.R., De Mendonca B., Bronstein M.D., Fragoso M.C.B.V. (2016). Negative correlation between tumour size and cortisol/ACTH ratios in patients with Cushing’s disease harbouring microadenomas or macroadenomas. J. Endocrinol. Investig..

[17] Witek P., Zielinski G., Szamotulska K., Maksymowicz M., Kaminski G. (2016). Clinicopathological predictive factors in the early remission of corticotroph pituitary macroadenomas in a tertiary referral centre. Eur. J. Endocrinol..

[18] Walia R., Dutta A., Gupta N., Bhansali A., Pivonello R., Ahuja C.K., Dhandapani S., Dutta P., Bhadada S.K., Simeoli C. (2021). Etiology-, Sex-, and Tumor Size-Based Differences in Adrenocorticotropin-Dependent Cushing Syndrome. Endocr. Prac..

[19] Greene L.W., Geer E.B., Page-Wilson G., Findling J.W., Raff H. (2019). Assay-specific spurious ACTH results lead to misdiagnosis, unnecessary testing, and surgical misadventure—A case series. J. Endocr. Soc..

[20] Findling J.W., Engeland W.C., Raff H. (1990). The use of immunoradiometric assay for the measurement of ACTH in human plasma. Trends Endocrinol. Metab..

[21] Nishioka H., Haraoka J., Akada K., Azuma S. (2002). Gender-related differences in prolactin secretion in pituitary prolactinomas. Neuroradiol..

[22] Aron D.C., Raff H., Findling J.W. (1997). EffectivenessVersusEfficacy: The Limited Value in Clinical Practice of High Dose Dexamethasone Suppression Testing in the Differential Diagnosis of Adrenocorticotropin-Dependent Cushing’s Syndrome. J. Clin. Endocrinol. Metab..

[23] Gibson S., Ray D.W., Crosby S.R., Dornan T.L., Jennings A.M., Bevan J.S., Davis J.R.E., White A. (1996). Impaired Processing of Proopiomelanocortin in Corticotroph Macroadenomas. J. Clin. Endocrinol. Metab..

[24] Shi J., Dhaliwal P., Zheng Y.Z., Wong T., Straseski J.A., Cervinski M.A., Shajani-Yi Z., Demarco M.L. (2019). An Intact ACTH LC-MS/MS Assay as an Arbiter of Clinically Discordant Immunoassay Results. Clin. Chem..

[25] Ikeda H., Yoshimoto T., Ogawa Y., Mizoi K., Murakami O. (1997). Clinico-pathological study of Cushing’s disease with large pituitary adenoma. Clin. Endocrinol..

[26] Pereira A.M., Van Aken M.O., Van Dulken H., Schutte P.J., Biermasz N.R., Smit J.W.A., Roelfsema F., Romijn J.A. (2003). Long-Term Predictive Value of Postsurgical Cortisol Concentrations for Cure and Risk of Recurrence in Cushing’s Disease. J. Clin. Endocrinol. Metab..

[27] Stroud A., Dhaliwal P., Alvarado R., Winder M.J., Jonker B.P., Grayson J.W., Hamizan A., Harvey R., McCormack A. (2020). Outcomes of pituitary surgery for Cushing’s disease: A systematic review and meta-analysis. Pituitary.

[28] Osamura R.Y., Grossman A., Korbonits M., Kovacs K., Lopes M.B.S., Matsuno A., Trouillas J. (2017). Tumors of the Pituitary Gland. WHO Classification of Tumours of Endocrine Organs.

